# Effect of Brominated Furanones on the Formation of Biofilm by *Escherichia coli* on Polyvinyl Chloride Materials

**DOI:** 10.1007/s12013-013-9578-8

**Published:** 2013-04-03

**Authors:** Ye Lianhua, Huang Yunchao, Xu Geng, Zhou Youquang, Zhao Guangqiang, Lei Yujie

**Affiliations:** 1 Cardiothoracic Surgery Section, Lung Cancer Research Center, Yunnan Institute of Oncology, No. 3 Affiliated Hospital of Kunming Medical University, Yunnan Province Tumor Hospital, Kunming, 650118 Yunnan China; 2 Cardiothoracic Surgery Section, Shangdong Heze Hospital, Heze, 274031 Shangdong China; 3 Clinical Bacteriology Laboratory, No. 3 Affiliated Hospital of Kunming Medical University, Kunming, 650118 Yunnan China

**Keywords:** Brominated furanone, Polyvinyl chloride, Bacterial biofilm, *Escherichia coli*

## Abstract

To study the influence of brominated furanones on the biofilm (BF) formation by *Escherichia coli* (*E. coli*) on polyvinyl chloride (PVC) material, and to provide new ways of surface modification of materials to clinically prevent biomaterial centered infection. Three brominated furanones, dissolved in ethanol, furanone-1(3,4-dibromo-5-hydroxyl-furanone), furanone-2(4-bromo-5-(4-methoxypheny)-3-(methylamino)-furanone), and furanone-3(3,4-dibromo-5,5-dimethoxypheny-2(5H)-furanone) with representative chemical structure, were coated on the surfaces of separate PVC materials (1 × 1 cm), respectively. The surface-modified PVC materials were incubated with *E. coli* and for controls, 75 % ethanol-treated PVC materials were used. This treatment played as control group. The cultivation incubations were for 6, 12, 18, and 24 h. The thickness of bacterial BF and bacterial community quantity unit area on the PVC materials was determined by confocal laser scanning microscopy (CLSM), and the surface structure of bacterial BF formation was examined by scanning electron microscopy (SEM). The results of CLSM indicated the thickness of bacterial BF and bacterial community quantity unit area on PVC materials treated with furanone-3 were significantly lower than that of control at all time points (*P* < 0.05), whereas, the differences between furanone-1 and furanone-2 groups and control group were not significantly different (*P* > 0.05). The results of SEM indicated that after 6 h incubation, the quantity of bacterial attachment to the surface of PVC material treated with furanone-3 was lower than the control group. By 18 h incubation there was completely formed BF structure on the surface of control PVC material. However, there was no significant BF formation on the surface of PVC material treated with furanone-3. The impact of different brominated furanones on SA biofilm formation on the surface of PVC materials are different, furanone-3 can inhibit *E. coli* biofilm formation on the surface of PVC material.

## Introduction

*Escherichia coli* is the common pathogenic bacteria in reparative and reconstructive surgery. *Escherichia coli* enters the body with biologic materials during reparative and reconstructive surgery, attaches at the surface of biologic material and forms bacterial BF [1–4]. Biofilm (BF) formation by bacteria on the surface of biomaterial is the basic reason for the multiple recurrent and uncured infections caused by biomaterial grafting and it usually causes significant damage [3–6]. Based on the establishment of the bacterial biofilm model on the surface of polyvinyl chloride (PVC) material in the previous study [1, 2], in the present study we examined how the surface coating of PVC by 3 kinds of brominated furanones influences the biofilm (BF) formation by *E. coli*. The results obtained provide novel ideas for the surface modification of biomaterials to prevent reparative and reconstructive biomaterial centered infection.

## Materials and Methods

### Experimental Material and Instruments

*Escherichia coli* reference stain ATCC25922, purchased from Culture collection center, Guangdong Institute of Microbiology. PVC (Guangdong Province Dongguan Kewei Medical Instrument Co., Ltd, Lot No.: 20050903); Furanone 1: 3,4-dibromo-5-hydroxyl–furanone (Fluka Company); Furanone 2: 4-bromo-5-(4-methoxypheny)-3-(methylamino)-furanone (Aldrich company); Furanone 3: 3,4-dibromo-5,5-dimethoxypheny-2(5H)-furanone (Aldrich company); Live/Dead^®^ Baclight™ Bacterial Viability Kit (Invitrogen); MRC-1024ES type confocal laser scanning microscope (CLSM, BIO-RAD); XL30ESEM type scanning electron microscopy (SEM, PHILIPS).

### Experimental Methods

#### Preparation of Bacterial Suspension

*Escherichia coli* frozen stock was inoculated in sterilized LB broth and incubated for 16 h at 37 °C and then plated on LB plate and incubated for 24 h (37 °C) to obtain colonies. Single colony was selected and inoculated in LB broth for 12–16 h (37 °C) to obtain *E. coli* suspension. The concentration of *E. coli* in the suspension was adjusted to 10^5^ CFU/ml for the experiment by pour-plate method [1].

#### Modification of PVC Surface and Experimental Groups

There were four groups of treatment: Control group, Furanone 1 group, Furanone 2 group, and Furanone 3 group. PVC was squared into 1 × 1 cm^2^ small pieces by the customized simulator and sterilized by fumigation with ethylene oxide. Brominated furanones, which are insoluble in water, were solubilized in ethanol and made up to 1 mg/ml solution. In Furanone groups, PVC was dipped in the corresponding ethanolic solution of furanone for 5 minto modify the surface of PVC piece. Then, the PVC piece was taken out of the solution and air-dried. In control group, PVC piece was dipped in 75 % ethanol solution for 5 min [7].

#### Formation of Bacterial Biofilm on the Surface of PVC

In 5 sterile flasks in each group, 20 ml LB broth, 5 μl bacterial suspension, and 4 corresponding PVC pieces were placed and the flasks were incubated at 37 °C. PVC pieces were taken out one after another, following incubations for 6, 12, 18, and 24 h (4 pieces for the observation by CLSM, another piece for the observation by SEM).

#### Sample Preparation and Observation of CLSM

The fluorescent stains for Bacterial viability on the BF was identified using Live/Dead^®^ Baclight™ Bacterial Viability Kit, according to manufacturer’s instructions; PVC pieces taken out of culture flasks were washed 3 times with distilled water and put into the fluorescent stains, stained for 20 min at room temperature. CLSM observation was with argon laser (514/488 nm), 1 visual field was randomly selected to observe the quantity of bacterial colony per field for each PVC piece observed. One bacterial biofilm captured randomly in every visual field was scanned from internal to external to measure its thickness.

#### SEM Sample Preparation and Observation

The PVC pieces were washed 3 times by HEPES buffer, pH 7, fixed on the object stage of SEM, dried in the critical CO_2_, the surface of PVC turned to the golden color due to the ions sputtering surface fixture deposition, the surface structure of bacterial biofilm was observed.

### Statistical Analysis

The data were analyzed by SPSS12.0 software, all the values were expressed as mean ± SD, the data were analyzed by repeated measure ANOVA, *P* < 0.05 was defined statistically significant.

## Results

### Effect of 3 Kinds of Furanones on the Formation of *E. coli* Colonies on the Surface of PVC Pieces

The CLSM examination revealed that the amount of bacterial colony (the quantity per unit area) on the surface of furanone 3 group PVC pieces was lower than that of control group (*P* < 0.05). There were no significant differences in the quantity per unit area of bacterial colony on the surface of PVC pieces of control group as compared with furanone 1 and furanone 2 groups (*P* > 0.05), see Table 1.

**Table 1:** Comparison of the quantity of Coli community on surface of PVC among groups (*n* = 4)

Time (h)	6	12	18	24
Control group	9.20 ± 0.66	11.60 ± 0.51	12.80 ± 0.86	15.80 ± 0.86
Furanones 1 group	8.60 ± 1.21	9.80 ± 1.16	12.00 ± 0.84	14.60 ± 1.03^#^
Furanones 2 group	9.40 ± 1.33	10.60 ± 1.12	13.00 ± 1.14	16.00 ± 1.22^#^
Furanones 3 group	4.80 ± 0.66*	6.80 ± 0.73*	7.60 ± 0.51*	8.80 ± 0.80*

* Compared with control group, *P* < 0.05

# Compared with control group, *P* > 0.05

### Effect of 3 Kinds of Furanones on the Thickness of *E. coli* Biofilm on the Surface of PVC Pieces

The results of the thickness of bacterial biofilm on the surface of PVC pieces measured by CLSM, indicated significantly less thickness in furanone 3 group than that of control group (*P* < 0.05). There were no significant differences in the thickness of *E. coli* biofilm on the surface of PVC pieces of control group compared with furanone 1 group and furanone 2 group (*P* > 0.05), see Table 2.

**Table 2:** Comparison of the thickness of *E. coli* community on the surface of PVC among groups (μm)

Time (h)	6	12	18	24
Control group	14.97 ± 1.01	28.23 ± 2.09	42.37 ± 2.26	75.06 ± 3.14
Furanones 1 group	14.40 ± 1.33	28.16 ± 2.66	41.73 ± 1.23	73.30 ± 3.99
Furanones 2 group	15.22 ± 1.29	26.82 ± 1.52	40.39 ± 1.30	74.66 ± 2.94
Furanones 3 group	10.95 ± 0.53*	20.92 ± 1.69*	27.88 ± 2.59*	37.09 ± 2.22*

* Compared with control group, *P* < 0.05

### Effect of 3 Kinds of Furanones on the Surface Structure of *E. coli* Biofilm on the Surface of PVC Pieces

**Fig. 1:**
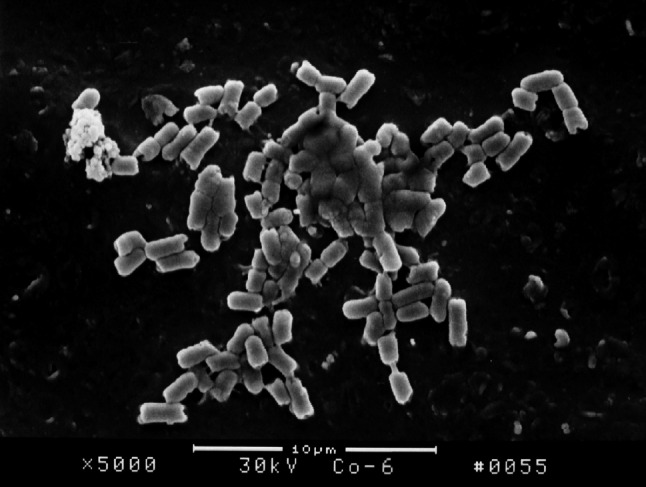
Examination of control group by SEM at 6 h

**Fig. 2:**
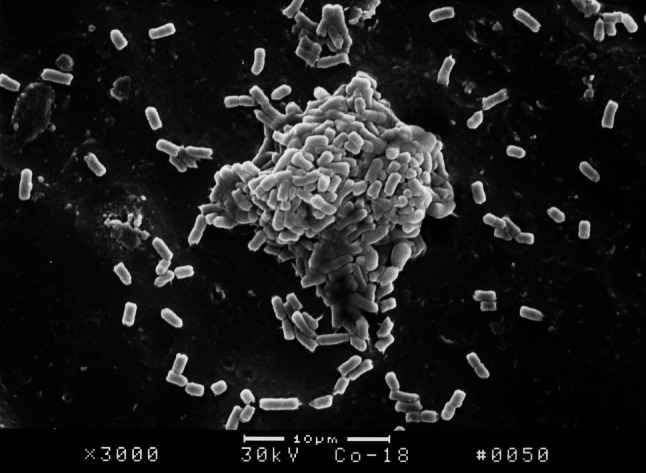
Examination of control group by SEM at 18 h

**Fig. 3:**
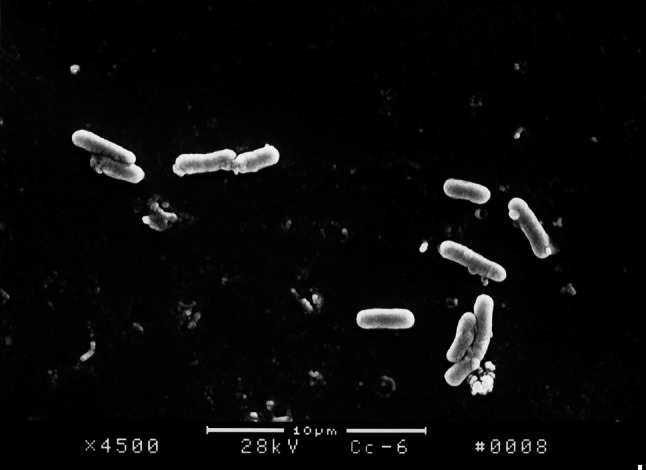
Examination of furanones-3 group by SEM at 6 h

**Fig. 4:**
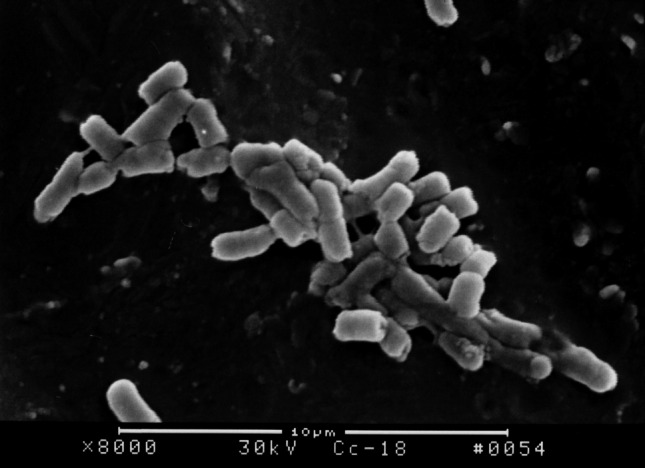
Examination of furanones-3 group by SEM at 18 h

**Fig. 5:**
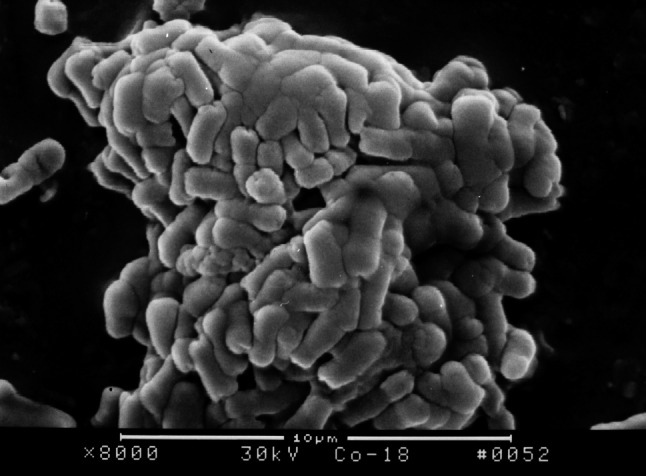
Examination of furanones-1 group by SEM at 18 h

**Fig. 6:**
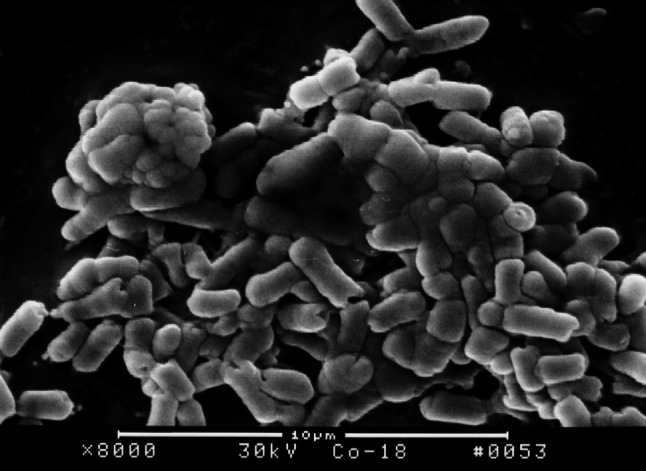
Examination of furanones-2 group by SEM at 18 h

The surface structure of *E. coli* biofilm on the surface of PVC pieces as observed under SEM indicated that a smaller quantity of bacterial colony attached on the surface of PVC pieces treated with furanone 3 compared with the control group. After incubation for 18 h, *E. coli* BF structure was fully formed on the surface of control group PVC material. However, there was no significant formation on the surface of PVC material treated with furanone-3, see Figs. 1, 2, 3, and 4. Similar to control group, there was obvious and significant BF formation in furanone 1 and furanone 2 groups at 18 h, see Figs. 5 and 6.

## Discussion

The formation of bacterial biofilm on the surface of biomaterial occurs in two phases, the initial attachment followed by aggregation [1, 2]. *Escherichia coli* first attaches on the surface of biomaterial, the initial attachment usually completed in 6–12 h. After the bacterial number on the biomaterial surface reaches a certain degree, the aggregation phase starts. In this phase, bacteria attach to each other, differentiate, proliferate and excrete a large amount of extracellular sticky substance. All the above come into being the highly organized structure of multi cell mass. As a result, bacterial biofilm forms in 18–24 h.

Quorum sensing plays a very important role in the formation of bacterial biofilm [7–10]. In quorum sensing, single bacterium releases autoinducers into surrounding environment. The autoinducer is a small molecule that transmits message to other bacteria. With the increased partial density of bacteria, the concentration of autoinducer also increase. When the concentration of autoinducer reaches a threshold value, the autoinducer’s receptor at the surface or interior of bacteria will be activated. The biochemical signal in the interior of bacteria is generated and induces the expression of specific genes in bacteria. Furthermore, the expression of these specific genes make bacteria to undergo stressful physiologic changes, such as DNA replication, formation of biofilm, generation of virulence factors, production of antibiotics, development of a sporophyte, and so on [10, 11]. This kind of gene regulation is called quorum sensing. A few studies reported that there are three kinds of quorum sensing which are related to the formation of bacterial biofilm: (1) Acylated homoserine lactones (AHL) quorum sensing exists in Gram-negative bacterium. Its autoinductor is *N*-acylhomoserine lactone (AHL). LuxI protein synthesizes AHL. AHL is synthesized from fatty acid and homoserine by LuxI protein. LuxR is the receptor of AHL, one of transcriptional regulatory proteins. AHL can freely pass through the bacterial cell membrane. When the concentration of AHL in the surrounding environment of bacteria reaches a threshold value, LuxR protein in bacteria is activated and AHL–LuxR complex forms. The transcription of target genes is initiated by the biochemical reaction in bacteria [11–13]. (2) Autoinducing peptides (AIPs) quorum sensing exists in Gram-positive bacterium. Its autoinducer is AIP, the amino acid or oligopeptide synthesized by bacterium. The concentration of AIPs that bacterium excretes into the exterior increases with increasing density of bacteria. When the concentration of AIPs reaches a threshold value, AlPs activate histidine protein kinase of the receptor, located in cell membrane and make it auto-phosphorylate. Then, histidine protein kinase phosphorylates responsive regulatory proteins. The phosphorylated responsive regulatory proteins act on the target genes and initiate the transcription and translation of target genes, including synthesis of AIPs [14, 15]. (3) Autoinducer-2 compounds (AI-2) quorum sensing distribute in Gram-negative and Gram-positive bacteria intensely. Autoinductor-2 (AI-2), a derivative of furanones, is the autoinductor of this quorum sensing. The bacteria synthesize AI-2 and excrete it to the exterior of bacteria. The concentration of AI-2 increases with the increasing density of bacteria. When the concentration of AIPs reaches a limiting value, AI-2 combines with luxP proteins in the periphery of cytoplasm. AI-2/luxP complex combines with histidine protein kinase in cell membrane, initiates multistage phosphorylation like AIPs and finally induces the expression of target genes [16, 17].

For each kind of quorum sensing, the molecular nature of autoinductor is not completely ascertained. Brominated furanones act on AHL and AI-2 principally, combine with the receptors competitively [14–17]. *Escherichia coli* has AHL and AI-2 simultaneously and brominated furanones produce an effect on both of AHL and AI-2 [11–13]. The data about the formation of bacterial mass in floating state [11–17] indicated that most of brominated furanones blocked quorum sensing of bacteria after combining with the receptors of autoinductors. However, some brominated furanones played a role of bacterial autoinductors and improved the formation of bacterial biofilm. Therefore, it will inhibit the formation of bacterial biofilm on the surface of biomaterial efficiently by interfering and blocking bacterial quorum sensing by appropriate brominated furanones.

Based on the establishment of the bacterial biofilm model on the surface of polyvinyl chloride (PCV) material in the previous study [1, 2], the surface coating of PVC was modified by 3 kinds of brominated furanones with representative chemical structure. To explore the influence of brominated furanones to the biofilm (BF) formation of *E. coli* on the PVC material. The results indicated 3,4-dibromo-5,5-dimethoxypheny-2(5H)-furanone had an inhibitory effect on *E. coli* colony quantity and colony thickness, however, 3,4-dibromo-5-hydroxyl–furanone and 4-bromo-5-(4-methoxypheny)-3-(methylamino)-furanone had no significant effect on the formation of *E. coli* BF. It is probably because 3,4-dibromo-5,5-dimethoxypheny-2(5H)-furanone combines with the receptors of autoinductors competitively and block *E. coli* quorum sensing, as a result, the formation of *E. coli* BF was restrained. The other two furanones cannot bind with the receptors of autoinductors, so they play no effect on the formation of *E. coli* BF. However, it is not clear whether 3,4-dibromo-5,5-dimethoxypheny-2(5H)-furanone acts on AHL or AI-2. It needs further research.
